# Deep Learning for the Assisted Diagnosis of Movement Disorders, Including Isolated Dystonia

**DOI:** 10.3389/fneur.2021.638266

**Published:** 2021-04-30

**Authors:** Syed Muhammad Arsalan Bashir, Yi Wang

**Affiliations:** School of Electronics and Information, Northwestern Polytechnical University, Xi'an, China

**Keywords:** dystonia, assisted diagnosis, movement disorder, deep learning, artificial intelligence

## Introduction

Functional movement disorder (FMD) is a disorder that is altered by distraction or non-physiological maneuvers (this includes excessive placebo response); this disorder is clinically different from movement disorders known to be caused by neurological disease (1). Dystonia is a movement disorder classified by involuntary patterned or twisting body movements, which further results in atypical postures (2). Isolated dystonia is a rare neurological disorder where the patient has dystonia without other neurological disorders like Alzheimer's. Dystonia often harms the quality of life and can be markedly disabling (3); furthermore, a relatively high number of cases are misdiagnosed or underdiagnosed because of the non-availability of a global biomarker.

In a recent research paper by Valeriani and Simonyan (4), a deep learning-based method was proposed, i.e., DystoniaNet, which can recognize a microstructural neural network biomarker for the diagnosis of dystonia from raw MRIs. The biomarker-based on DystoniaNet depicted an overall accuracy of 98.8%, including 3.5% cases in which the network referred the case for further analysis with a diagnosis time of 0.36 s per subject; this was a significant improvement over shallow machine learning networks. The assisted diagnosis of isolated dystonia using deep learning has opened a new direction where computational intelligence plays its part in the early diagnosis of movement disorders.

## Discussion

**Figure 1:**
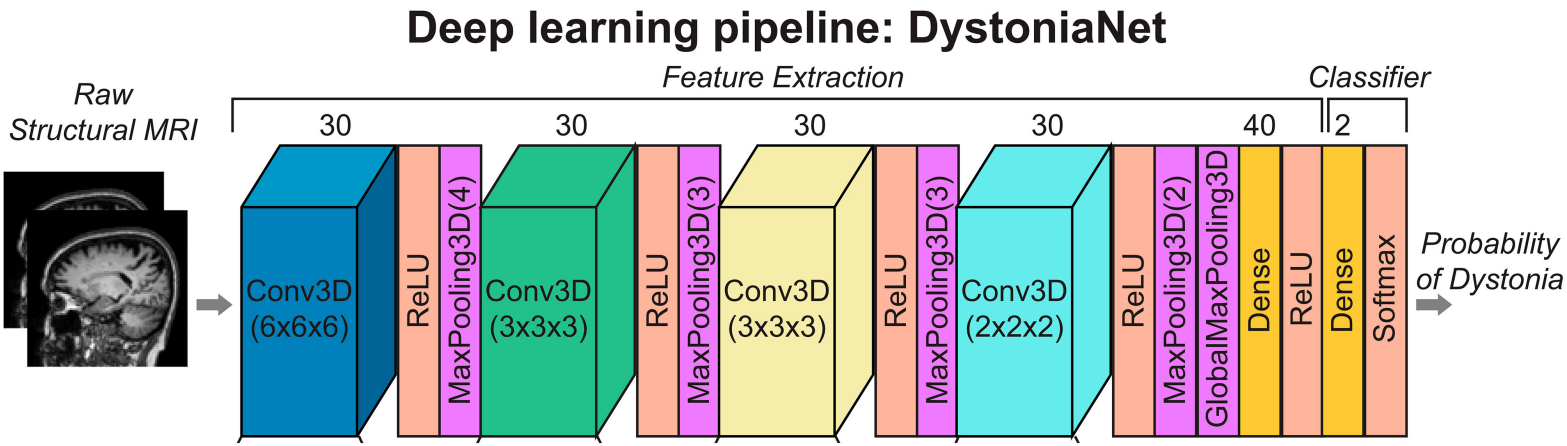
Deep learning and shallow machine-learning pipelines for diagnosis of isolated dystonia. Raw structural brain MRIs were used with the deep learning pipeline (DystoniaNet). Copyright (2020) National Academy of Sciences.

DystoniaNet pipeline utilizes four convolutional layers (Conv3D), each followed by the rectified linear unit (ReLU) activation and maximum pooling (MaxPooling3D) layers; furthermore, there are two densely connected layers, one for feature extraction and another for classification, which is also the output layer (see Figure 1).

Comparing the performance of DystoniaNet with the current state-of-the-art methods is essential in benchmarking the method for the universal classification of dystonia. The choice of benchmarking the proposed DystoniaNet (which is a deep neural network-based approach) with linear discriminant analysis (LDA) and support vector machine (SVM) was justified as these methods showed promising results in the classification of laryngeal dystonia and cervical dystonia (5, 6). In contrast, using a single-layer artificial neural network (ANN) seems to be an odd choice as there are methods similar to DystoniaNet, which share a similar network pipeline, for example, VGG and VoxCNN. Selecting appropriate methods to compare and contrast any deep learning-based diagnosis is critical because it provides insights into the performance of various methods under similar settings (dataset, computational setup, and programming environment).

Since its conception, deep learning methods have been used in medical data analysis; for an overview of deep learning applications in medical data analysis, we recommend readers to go through the review papers by Jang and Cho (7) while Zhang et al. (8). In the past decade, deep learning has been employed in classification, segmentation, and identification tasks in assisted or fully automated diagnosis of brain-related disorders. Oh et al. (9) in 2020 used 13 stacked convolutional neural networks (CNNs) to build an automated system for the detection of Parkinson's disease (PD) with an accuracy of 88.25%, while Bakiya et al. (10) and Khamparia et al. (11) used deep learning-based methods for the assisted diagnosis of neuromuscular disorders.

Simonyan and Zisserman (12) proposed a very deep convolutional neural network called VGG for large-scale image classification and introduced small convolution filters of the size of 3 × 3 to design very deep convolutional networks (up to 19 layers). The proposed pipeline of VGG was further used by Korolev et al. (13) to design 3D convolution-based VoxCNN, which successfully classified Alzheimer's disease vs. mild cognitive impairment and normal controls using brain MRI scans. The pipeline of VoxCNN [Refer to Figure 1 in Korolev et al. (13)] is closely related to DystoniaNet as VoxCNN had four volumetric convolutional blocks for extracting features, two dense layers, and a dense output layer with softmax non-linearity for classification (13). Valeriani and Simonyan (4) used the deep learning pipeline structure similar to Korolev et al. (13), which shows the applications of deep learning in the assisted diagnosis of movement disorders, especially those that do not have a global biomarker.

Shallow networks like a single layer ANN with only six neurons in the hidden layer can only extract limited features discussed by Valeriani and Simonyan (4) in the supplementary data file. Additionally, shallow methods performed adversely while using the input features from DystoniaNet. Islam and Zhang (14) used brain MRI data for multi-class classification of Alzheimer's disease using an ensembled model and deep neural networks to achieve multi-class accuracy of 93.18%. It would be interesting if performance evaluation of DystoniaNet is performed against the methods mentioned above, like VoxCNN (13), Ensembled method (14), especially when using DystoniaNet identified input features as input since these are the state-of-the-art methods used for classification of FMDs and have already been used for the assisted diagnosis of movement disorders. Furthermore, DystoniaNet introduced a new research direction where further investigation is required to apply VGG or VoxCNN (13), Ensemble method (14), and other deep learning-based methods to learn the microstructural biomarkers for the classification of dystonia and other FMDs.

When comparing deep learning-based methods for diagnosis in medical data, it is essential to compare existing state-of-the-art methods in terms of accuracy of the model, computational efficiency, and inference time. Although the most crucial parameter is the overall accuracy of prediction, computational efficiency depicts the computational resources required for a given deep learning model. The inference time is critical because it conveys the prediction time of a method using the pre-trained parameters. Another critical factor for a deep learning method is the number of parameters; for instance, VGG-A (12) generated 133 million parameters on the ImageNet large-scale visual recognition challenge (ILSVRC). Valeriani and Simonyan (4) did not share the model's parameters, making it difficult to compare the proposed method's computational efficiency to other state-of-the-art methods. For DystoniaNet, the inference time was 0.36 s per subject, which should be compared to other state-of-the-art methods.

## Conclusion

In summary, it is crucial to validate the performance of deep learning-based assisted diagnosis methods with the existing state-of-the-art methods. The use of deep learning-based methods for the assisted diagnosis of dystonia is a promising application of deep learning in movement disorders; however, there is a need to introduce cross-subject validation by subject experts to ensure the evaluation and comparison is performed with the existing state-of-the-art methods. This opinion paper highlights the research gaps in assisted diagnosis of movement disorders while providing future researchers an opportunity to apply deep learning-based methods for classification, identification, and diagnosis of brain-related disorders, including functional movement disorders.

## Author Contributions

SMAB: conception, analysis, interpretation, and manuscript writing. YW: supervision and review. All authors approved the final manuscript.

## Conflict of Interest

The authors declare that the research was conducted in the absence of any commercial or financial relationships that could be construed as a potential conflict of interest.
